# Factors Influencing Work–Life Balance in Physicians and Advance Practice Clinicians and the Effect of Heartfulness Meditation Conference on Burnout

**DOI:** 10.1177/2164956118821056

**Published:** 2019-01-15

**Authors:** Jayaram R Thimmapuram, Rodney Grim, Theodore Bell, Ronald Benenson, Mark Lavallee, Mihir Modi, David Noll, Ridgley Salter

**Affiliations:** 1 Department of Internal Medicine, WellSpan Health, York, Pennsylvania, USA; 2 WellSpan Emig Research Center, WellSpan Health, York, Pennsylvania, USA; 3 Thomas Hart Family Practice Center, WellSpan Health, York, Pennsylvania, USA; 4 WellSpan Family Medicine – Dallastown, WellSpan Health, York, Pennsylvania, USA; 5 WellSpan Medical Group Administration, WellSpan Health, York, Pennsylvania, USA; 6 WellSpan Family Medicine – Hayshire, WellSpan Health, York, Pennsylvania, USA

**Keywords:** stress, health care, coping, yoga

## Abstract

**Background:**

Burnout levels have risen in recent years and satisfaction with work–life balance has decreased. Individual and organizational factors may affect burnout in physicians and advance practice clinicians (APCs). Meditation is a tool to mitigate stress and enhance well-being. In this study, we assessed the factors affecting work–life balance in physicians and APCs. We also measured the impact of Heartfulness meditation conference on burnout.

**Methods:**

Physicians and APCs were surveyed through an abbreviated Maslach burnout inventory (aMBI) to assess the burnout levels and a question to assess the factors influencing work–life balance. Wellness initiatives included either attending a Heartfulness meditation conference or reading a book about burnout and wellness (approximated at a 3-h read). A repeat aMBI survey was sent 8 weeks after the conference. Pre- and postburnout scores were assessed.

**Results:**

Of the 1393 physicians and APCs, 537 responded to the aMBI, and there were 414 comments (663 factors) for the question on work–life balance. Among the respondents, 60.5% and 32% had symptoms of moderate to severe emotional exhaustion (EE) and depersonalization, respectively. Twenty-eight percent of the respondents had symptoms of moderate to low personal accomplishment. The major factors impacting work–life balance included work load, work flow, and scheduling. A follow-up aMBI survey was completed by 79 from the conference group and 264 from the nonconference group. In the age-group between 30 and 50 for the conference group (n = 40), mean EE decreased from 9.8 to 8.6 with statistical significance (*P* = .014). There was no statistically significant change in the nonconference group in any age-group.

**Conclusion:**

Workload, workflow, and scheduling issues were the major factors affecting work–life balance. There is a significant level of burnout in physicians and APCs. Heartfulness meditation conference was associated with a significant decrease in EE in those aged 30 to 50 years. There was no significant change seen in the nonconference/book reading group.

## Introduction

Physicians have a high prevalence of depression and burnout.^1–3^ Exposure to severe and chronic stressors may contribute to higher levels of burnout leading to deleterious consequences such as depression, anxiety, substance abuse, marital dysfunction, premature retirement, and suicide.^4,5^ Nurse practitioners and physician assistants (advanced practice clinicians [APCs]) also report high levels of stress.^6,7^ Interventions at individual-focused and organizational levels have been shown to reduce burnout in physicians.^8^ The Maslach Burnout Inventory (MBI), the most widely used tool for measuring burnout, has been validated for multiple populations.^9,10^ Abbreviated MBI (aMBI) is a 9-item version of the MBI with 3 short form subscales in each dimension of burnout: emotional exhaustion (EE), depersonalization (DP), and personal accomplishment (PA). This has been shown to be valid and can be used to rapidly screen group of employed health-care practitioners for symptoms of each of the 3 dimensions of burnout.^11^

Physicians in the United States are reported to experience higher levels of EE when faced with work–life conflict.^12^ Burnout and satisfaction with work–life balance in U.S. physicians worsened from 2011 to 2014. More than half of U.S. physicians have symptoms of professional burnout.^13^ A wide range of strategies have been used to combat burnout and maintain a balance in life including conferences, retreats, self-care, providing resources through books, spending time with family, limitation of work hours, and tools for positive outlook.^5^ Meditation is a tool with potential for reducing stress and improving wellness that has been studied in the literature. Techniques such as yoga and meditation have been shown to decrease exhaustion, stress, fatigue, and burnout.^14^ Heartfulness meditation offered by Heartfulness Institute (www.heartfulnessinstitute.org) is a simple, easy to implement, heart-based meditation practice aimed at achieving a state of balance of mind. It has been shown to decrease burnout in physicians and nurses.^15^ Research suggests that organizational and individual-focused interventions may be necessary to reduce burnout.^8^

This article explores this unique combination of organizational initiative facilitating the individual-focused intervention.

### Objective

In this organizational initiative, the factors impacting work–life balance and prevalence of burnout in physicians, nurse practitioners, and physician assistants from 4 hospitals of a large health-care organization were studied. The impact of wellness initiatives on burnout was assessed through aMBI. Initiatives included attending a Heartfulness meditation conference or reading 1 of 3 books about burnout and wellness (approximately a 3-h read) chosen by the organization’s learning resources department.

## Methods

The year of the initiative is the academic year 2017–2018.

### Overview

The study was conducted at a large, East Coast-based health-care organization with 1393 physicians and APCs across 4 hospitals. This was categorized as an innovative quality improvement project and was deemed “not human subjects research” by the organization’s institutional review board. The wellness initiative involved assessing the factors impacting work–life balance and measuring a baseline organizational burnout prevalence. Factors impacting work–life balance were assessed through an anonymous open-ended question (“How can we as an organization help you have a ‘better balance’ in your life?”) on what impacted respondent’s work–life balance. Burnout was assessed through an anonymous aMBI e-mail survey. Physicians and APCs voluntarily self-assigned to participation in either a Heartfulness meditation conference or reading a book on wellness and burnout, which constituted the nonconference group. Participation in either arm of the study was included as part of a financial compensation plan. Since either choice was part of the compensation plan, it was unlikely the selection contributed to bias. Burnout outcomes were evaluated by a repeat aMBI survey 8 weeks post conference. Pre- and postburnout scores were assessed.

aMBI is a shorter version of MBI that has been validated to assess the 3 primary dimensions of burnout. EE is further classified as indicative of low (≤6), moderate (7–10), or high (≥11) level burnout. The scores of ≤3, 4 to 6, and ≥7 are indicative of low-, moderate-, and high-level burnout for DP. For PA, low-, moderate-, and high-level burnout for personal accomplishment are indicated by the scores of ≥15, 13 to 14, and ≤12, respectively.

### Data Analysis

Basic descriptive statistics of the study groups were performed. Changes in aMBI scores between the conference group and the nonconference group with the alternative activity of reading a book were analyzed by paired-sample *t* test. α < .05 were considered statistically significant. Statistics were calculated using SPSS v.24 (IBM, Armonk, NY).

### Intervention

#### Conference group:

All participants assigned to the conference group received an overview of the conference and attended the sessions conducted by Heartfulness trainers. Each session included a presentation followed by a practical guided meditation–related activity. The topics included

1. “Introduction to Heartfulness” followed by a guided activity of relaxation and meditation.
2. “Fostering Nurturing Environment” followed by a guided activity of Heartful Affirmations.
3. “From Burnout to Joy” followed by a guided activity of Rejuvenation.

Each session was for 1 h. Participants were provided written instructions on Heartfulness meditation to continue the practice at their convenience.

#### Nonconference group:

A choice of books on wellness and burnout were provided with the idea of promoting understanding about burnout and developing strategies to cope with burnout. These books included:

1. Burnout: The Cost of Caring, by Christina Maslach.
2. When Breath Becomes Air, by Paul Kalanithi.
3. The Compassion Fatigue Workbook, by Francoise Mathieu.

## Results

### Response Rate

**Figure 1.**
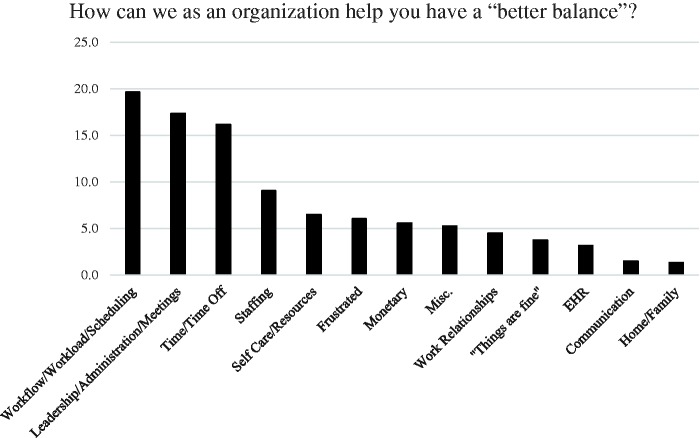
Factors influencing work-life balance.

A total of 1393 surveys were sent out. Of these, 530 respondents gave complete baseline data, and 343 responded to the follow-up survey. Of these 343, 79 (23.1%) were in the conference group and 264 (76.9%) were in the nonconference group. There were 40 (22.3%) people aged 30 to 50 years in the conference group, and 139 (77.7%) people aged 30 to 50 years were in the nonconference group. Work–life balance: There were 414 individual comments, which included 663 factors on work–life balance. The major factors impacting work–life balance included work load, work flow, and scheduling, followed by comments on administration and meetings, time/time off, staffing, and self-care (Figure 1).

Sample comments by category include the following:

#### Workload/workflow/scheduling

- Avoid adding more work within the time frame that is unrealistic to accomplish.
- I love seeing patients. I become overwhelmed at the end of the day when I am faced with so many tasks and phone calls.
- Reassess the amount of time we spend before hours, after hours, nights and weekends at home preparing or completing charts, reviewing tasks, etc.
- Never ask Drs to do one more clerical duty, and get rid of the multiple clerical duties we are already tasked with.
- Less data entry, more patient care. I’ve become an excellent data entry clerk but lost my empathy.
- Presently I spend at least 75% of my day doing things that don’t require a medical degree.

#### Leadership/administration/meetings

- Eliminate/reduce directives/initiatives that are not evidence based.
- Appreciation and respect for the work that I do.
- Improving the employee retention program, rather than letting go good employees.
- Give us the tools to make nonpatient time more efficient. Continue to refine EHR and improve our ability to use it as a tool to be more efficient.
- Make sure meetings are for a good reason and well run.

#### Staffing

- Better staffing so that I don’t leave work as exhausted and overworked.
- Adequately provide staff to cover when one team member is on vacation. Too much burden is left to those at work too frequently.
- I feel guilty when taking off. Coverage is an issue as staff get overwhelmed when trying to cover two positions.
- More support staff. I feel providers have too much on their plate. More nurses at the hospital and more support staff in the office would help off load this.

#### Time and Time off

- Respecting time off. Be more respectful of physician’s time. Realize that every time that you add another computer-based data entry task in a doctor’s schedule that this encroaches on their ability to be a doctor and enjoy their job. Even though we are all employed, it would be nice to feel as though we are the primary priority of the health system.
- Reassess the amount of time we spend before hours, after hours, nights and weekends at home preparing or completing charts, reviewing tasks, etc.
- Every effort should be made to avoid allowing work-related activities creep into physician’s personal time. We should avoid having any meetings, training sessions, or other required attendance events outside of normal working hours.
- Incentive to use your vacation days.

#### Self-care/resources

- Meditation space, reciprocate goodness, empower each other.
- Ease of taking time off for “mental health” days.
- More work–life flexibility (ie, feasibility of off-premise care, reimbursement for time regardless of location), large and accessible exercise/gym on-premises.
- Have programs/courses that address work/life balance.

#### Relationships and communication

- I enjoy my job and enjoy working. I wish I could optimize my daily schedule with my home life (children’s school and sports). I’m often at the office late so I don’t get to take my child to her activities or do homework with her.
- The paperwork portion is what gets to me. I love seeing patients, but after I have poured out with them for 8–9 hours, knowing I have 1–2 hours of paperwork following can be tiring. Sometimes I end up bringing work home. And that really can affect my home life.
- More organized activities to interact socially with coworkers (clinicians and providers).
- Not sure. We need to be more creative. I was just off and came back to 150 e-mails and 40 patient messages. I knew this was hanging over me and it was hard to relax because of this. This does not include the other things which need to be addressed.

#### Some other significant comments

- The medical industrial complex is an entity that has crushed many a doc.
- Not sure! I have been a care giver most of my life and feel as though I am done.

### Baseline Organizational Burnout

**Figure 2.**
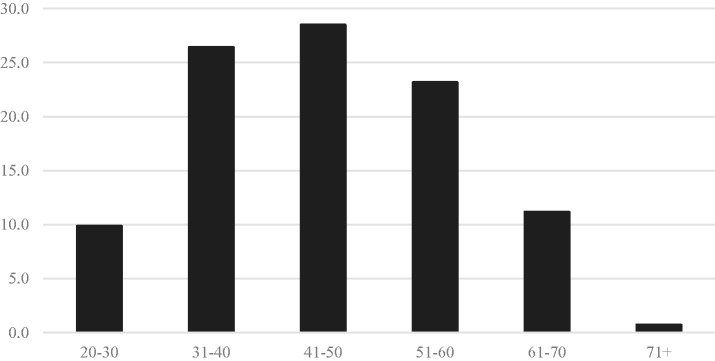
Age distribution in percentage.

**Figure 3.**
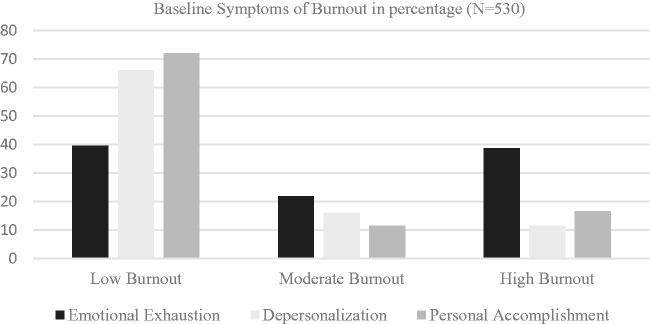
Burnout symptoms in percentage.

**Figure 4.**
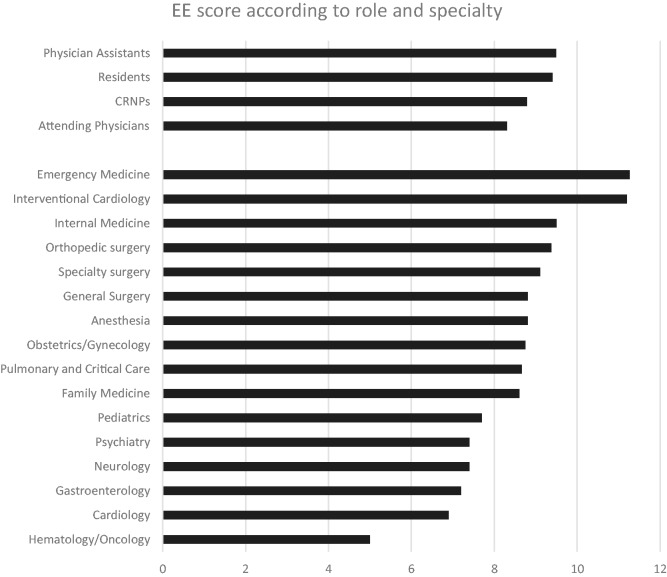
Emotional exhaustion score according to role and specialty.

In total, 530 of 1393 physicians and APCs responded to the initial aMBI. Fifty-four percent were female; 25.3% were hospital based, 43.9% were office based, and the remaining were both office and hospital based. The distribution according to age is shown in Figure 2. Among the respondents, 60.5% and 32% had symptoms of moderate to severe EE and DP, respectively. Twenty-eight percent of the respondents had symptoms of moderate to low personal accomplishment (Figure 3). Mean EE scores according to the role and specialty are shown in Figure 4. Only the specialties with 5 or more responses were included.

### Meditation Conference Versus Nonconference Group

**Figure 5.**
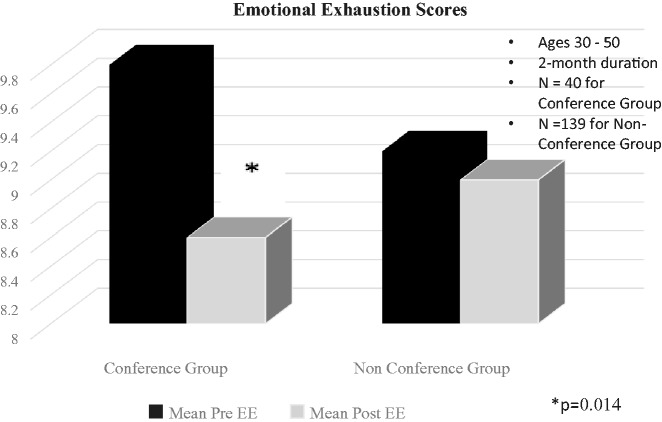
Emotional exhaustion scores in conference and non-conference group.

A follow-up aMBI survey was completed by 79 participants of the conference group and 264 from the nonconference group. Overall, there were no significant changes in burnout dimensions in either group. However, the conference group attendees had a greater reduction in the mean EE from 9.8 to 8.6 in the age-group between 30 and 50, with statistical significance (n = 40; *P* = .014). Mean pre- and post-EE scores in the nonconference group for this age-group were 9.2 and 9.0, respectively (n = 139; *P* = .675; Figure 5). This age-group constituted over 50% of the physicians and APCs who attended.

## Discussion

The main factors impacting work–life balance, thus potentially burnout, included workload, work flow, and scheduling issues. The present initiative supports the contention that U.S. physicians report a high rate of burnout. Although previous reports suggest that about 50% of U.S. physicians indicate burnout,^13^ the results of this survey found a slightly higher incidence at 60%. Emergency medicine, interventional cardiology, and internal medicine specialties showed high degree of EE in this study. Physician assistants and residents compared to nurse practitioners and attending physicians had a higher score of EE.

Interventions in which physicians in the local work environment are engaged in design and implementation of wellness strategies may reduce burnout.^16^ Although it is important to reduce stress at the individual level, work processes and improving the fit between the organization and the individual physician are also crucial for tackling burnout.^12^ The interventions involved in this article were chosen by the compensation committee of the organization involving physicians from various specialties. This was an initial step toward increasing awareness and providing some tools to improve wellness.

Previous research suggests that the age of the physicians also plays a significant role. Younger physicians are at increased odds of burnout, with those who are younger than 55 years at 200% increased risk compared to those older than 55 years.^17,18^ In this study, age played a role in response to interventions. Participants 30 to 50 years of age had a higher favorable response to Heartfulness meditation conference. This age-group made up most of the study group, constituting over 50% of the physicians and APCs.

On an individual level, a variety of personal wellness strategies, including exercise, focusing on what is important personally in life, taking vacations, and nurturing one’s religious/spiritual life have all been identified as protective against burnout.^19^ At an organizational level, some health-care systems have launched wellness programs that promote resilience improvements and burnout reductions through a variety of initiatives and programs including online resources, stress reduction and mindfulness classes, and a crisis hotline.^20^ Adequate staffing, good leadership, and support were found to reduce the risk of burnout.^21^ A 12-week nonintensive Heartfulness meditation program was shown to improve burnout and emotional well-being.^15^ A mindfulness education program involving an 8-week intensive phase, followed by a 10-month maintenance phase was found to be helpful in mitigating burnout.^22^ Stress management programs were found to have limited evidence of benefit, and the effects were found to diminish without booster sessions.^23,24^

Meditation conference was associated with a sustained reduction of EE in mid-career physicians and APCs at 8 weeks post conference. The results of these initiatives add to the body of evidence supporting organizational promotion of individual practices such as meditation to help reduce burnout. Training in Heartfulness meditation is a simple yet effective method to support physicians and APCs. It can equip them with self-practice tools and techniques to improve wellness and decrease burnout.

### Limitations

This study had some limitations. The nonconference group had an option of reading a book on wellness and burnout; however, it is not possible to determine if participants read it. Also, this study is relational and cannot determine cause and effect. Self-selection to either group prevents randomization. aMBI is valid and reliable, but it is not the same gold standard as the full version of the MBI. This study did not measure a dose-response to the intervention group. Meaning, the meditation participants were not asked how often they were engaging in meditation (times/week) to determine if there was a certain dose of meditation needed to show improvement in EE. People who chose to attend the conference may have been more likely be compliant with the program and therefore the benefit. We chose 2 months as a follow-up time. Although a difference may be ascribed to the intervention, the intervention could have lost effectiveness in the 2-month period that was not detected. Other factors, such as health-care environment, which could impact results, were not evaluated.

### Conclusion

There is a significant level of burnout in physicians and APCs noted in our specific study population. Workload, workflow, and inadequate staffing were reported to be the major factors affecting work–life balance. Heartfulness meditation conference attendance was associated with a significant decrease in EE with statistical significance in mid-career professionals aged 30 to 50 years. The impact was sustained at 2 months. There was no significant change seen in the nonconference group. A takeaway of this project is that an organizational offering of meditation programs should be supported and can offer providers with a useful coping skill to combat stress/burnout. Organization-initiated meditation conferences could be a potential option to engage physicians and APCs and improve well-being. Although meditation may not be for everyone, offering it to providers as one of several approaches to stress and burnout should be considered. The work–life balance responses identify many underlying organizational issues. We recommend further studies to enhance the evidence.
